# Disseminated histoplasmosis from western Mexico—rethinking our geographic distribution of endemic fungal species: a case report and review of literature

**DOI:** 10.1186/s13256-024-04856-x

**Published:** 2024-11-08

**Authors:** Richard Sleightholm, Daniel Z. Hodson, Isabella So, Harshika Avula, Jagmohan Batra

**Affiliations:** 1 Department of Pediatrics, David Geffen School of Medicine, University of California, Los Angeles, CA USA; 2 Division of Internal Medicine-Pediatrics, David Geffen School of Medicine, University of California, Los Angeles, CA USA; 3 Division of Pediatric Infectious Diseases, Memorial Care Miller Children’s & Women’s Hospital Long Beach, Clinical Professor of Pediatrics, University of California Irvine Department of Pediatrics, Irvine, CA USA

**Keywords:** Mycoses, Disseminated histoplasmosis, *Histoplasma capsulatum*, Chicken reservoir, *Histoplasma* endemicity in Mexico, Global health

## Abstract

**Background:**

*Histoplasma* is a fungal pathogen found in many parts of the world. In North America, its distribution is traditionally thought to be endemic to the Ohio and Mississippi River valleys. Development of histoplasmosis after *Histoplasma* exposure is related to degree of inoculum exposure and susceptibility, for example, immunocompromised status. Most exposed, healthy individuals are asymptomatic and few develop pulmonary symptoms. A limited number of infectious etiologies (that is, *Histoplasma*, *Coccidioides*, and *Mycobacterium tuberculosis*) can cause miliary pattern on chest imaging, and thus, histoplasmosis should be considered whenever a patient presents with pulmonary symptoms and these unique radiographic findings.

**Case presentation:**

A previously healthy 13-year-old Hispanic male presented as a transfer from an outside hospital with fever and hypoxia in the setting of a progressive, subacute gastrointestinal illness. Given hypoxia, the concern for sepsis, and unclear etiology of his illness, broad-spectrum antimicrobial therapy and noninvasive ventilation were started. Initial evaluation demonstrated miliary pulmonary infiltrates, and travel history raised suspicion for coccidioidomycosis or tuberculosis. After a complete evaluation, lab studies confirmed a diagnosis of histoplasmosis, and the patient made a full recovery after the initiation and completion of antifungal therapy.

**Conclusion:**

Herein, we present a patient who acquired histoplasmosis from an area of Mexico not currently acknowledged as endemic and review recently published data emphasizing new areas of *Histoplasma* endemicity in North America, particularly the southwest USA and most states of Mexico. Though limited surveillance data exist, mounting case reports/series and local epidemiologic studies illustrate the expanding worldwide endemicity of *Histoplasma* and underscore histoplasmosis as a growing global health concern.

**Supplementary Information:**

The online version contains supplementary material available at 10.1186/s13256-024-04856-x.

## Introduction

Histoplasmosis results from inoculation and infection by the dimorphic fungus *Histoplasma* spp. Two distinct variants have traditionally been described, *Histoplasma var. capsulatum* in the Americas and *Histoplasma capsulatum var. duboisii* in sub-Saharan Africa [1]. Recently, four genetically distinct species of *Histoplasma* have been identified [2]. Inoculation occurs from the disruption and aerosolization of spores. Histoplasmosis is classically associated with spelunking/cave exploration and guano exposure. However, more pedestrian exposures may occur from both small-scale (for example, cleaning chicken coops) and large-scale (for example, construction zones) activities that disrupt soil contaminated with bird feces [3]. Epidemiologic studies have further expanded our understanding of inoculum exposure to occupations involving dusty conditions, bird excrement handling or processing, fertilizers containing bat guano, and poultry farming [4, 5]. Most healthy individuals may not experience symptoms after exposure to *Histoplasma*, while others may develop mild to moderate pulmonary disease with symptoms of fever, headache, myalgia, cough, and chest pain, often mistaken for community-acquired pneumonia [6]. More atypical presentations include rheumatologic complaints, cutaneous manifestations, mediastinal granulomas, or fibrosing mediastinitis with risk of local mass effect and disseminated disease with neurologic or visceral involvement [7–12]. The development of clinical disease and degree of severity are related to a number of factors including immune status, extent of inoculum exposure, and genetic susceptibility to mycoses, specifically dysfunctional interferon-γ-mediated immunity (e.g. Mendelian susceptibility to mycobacterial disease, STAT1 gain-of-function, interleukin-12Rβ1 deficiency, interferon-γ receptor 1 deficiency, or GATA2 deficiency) [13–16]. Patients with one or more of these predisposing factors may develop acute severe (diffuse) pulmonary disease and progress to extrapulmonary manifestations and disseminated disease [6]. Unfortunately for some individuals, acquiring histoplasmosis can result in severe morbidity or even mortality. Reducing the risk of contracting this illness is paramount, but this relies heavily on current epidemiological data to help make informed recommendations. Histoplasmosis has been linked to Central America since the turn of the twentieth century [17], but epidemiologic risk has traditionally been considered to be low in the southwest USA and western Mexico.

The case presented herein describes a previously healthy teenage male who traveled to an ostensibly low-risk area of Mexico but contracted disseminated histoplasmosis while visiting a chicken farm. A detailed review of our patient’s exposure history, a thorough examination of the current literature, and discussions with local infectious disease experts in Mexico revealed that our patient’s histoplasmosis infection was not a unique occurrence, and *Histoplasma* endemicity in western Mexico is known to local infectious disease experts with published and unpublished cases from western Mexico lending credence to the region likely being endemic for *Histoplama*. However, this knowledge is not readily available to healthcare providers globally, and exposure risk resources such as those listed on the Centers for Disease Control and Prevention (CDC) website do not reflect the expanding areas at risk. Thus, this case underscores the current discrepancy between local expertise of *Histoplasma* endemicity and internationally accessible resources on risk of exposure. Furthermore, accumulating reports are similarly highlighting expanding *Histoplasma* endemicity in other continents raising further concern that this global fungal infection constitutes a reemerging pathogen.

## Case report

A previously healthy, 13-year-old Hispanic male presented to an outside emergency department for > 1 month of tactile fevers, chills, cough, diarrhea, decreased appetite, fatigue, and lethargy. The patient was noted to be febrile to 39.2 °C, tachycardic to 152 beats per minute, tachypneic with respiratory rate of 22 breaths per minute, and hypoxic on room air (92% SpO_2_), prompting the administration of supplemental oxygen via nasal cannula. Labs were notable for transaminitis with aspartate aminotransferase (AST) 130 U/L and alanine aminotransferase (ALT) 52 U/L. Chest radiograph (CXR) showed diffuse interstitial thickening and nodularity concerning for atypical pneumonia. He received intravenous (IV) ceftriaxone 2 g and azithromycin 500 mg. Due to multisystem organ involvement and concern for sepsis, the patient was transferred to our institution for a higher level of care. Upon arrival, the patient was tachypneic to >30 breaths per minute with normal oxygen saturation on 1 L O2 via nasal cannula and hypertensive at 117/90 mmHg. The physical exam was notable for mildly coarse breath sounds bilaterally.

About 5 weeks prior to presentation, the patient had traveled to visit family in a small town located 100 km southeast of Guadalajara, a major metropolis in western Mexico. Prior to the trip, the patient and all immediate family members were in their usual good state of health, but his aunt in Mexico had a febrile illness with a cough.

Shortly after arrival to Mexico, several family members developed a febrile illness that lasted only 1–2 days. The patient denied any illness while in Mexico. Possible infectious exposures included visiting a waterpark, exposure to farm animals (goats, rabbits, and chickens), and ingestion of unknown but fully cooked meat products. The patient and family denied consumption of unpasteurized dairy products, cave exploration, bird or bat exposure, and insect or tick bites. However, further questioning revealed that during the trip, the patient had been inside a very large and dusty chicken coop that was being cleaned during windy weather conditions.

Upon returning to the USA, the patient subsequently developed fever, decreased appetite, and nonbloody, watery diarrhea. Other family members developed similar gastrointestinal (GI) symptoms, which resolved within a few days. Our patient continued to experience diarrhea with at least three episodes daily. Eating also elicited postprandial abdominal and chest pain, resulting in minimal food intake and  > 7 kg weight loss since his return from the trip. The patient also endorsed daily tactile fevers with facial flushing and sweating since his return. Subsequently, he developed a productive cough, fatigue, and altered mentation.

**Fig. 1:**
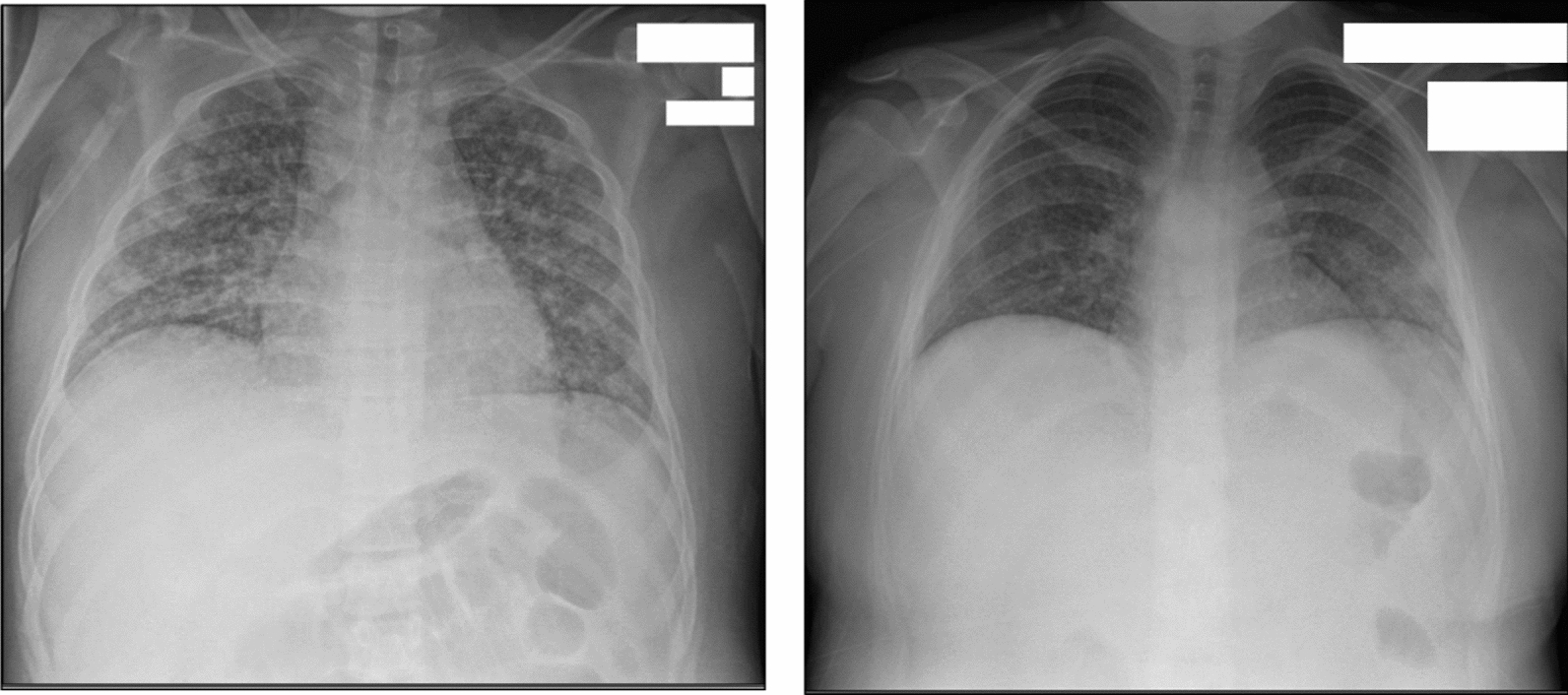
Initial chest radiograph of the patient on admission (left) showing diffuse bilateral heterogeneous infiltrates. After completion of intravenous liposomal amphotericin B and transition to posaconazole, the patient’s respiratory status improved with repeat chest radiograph (right) showing marked reduction in infiltrative pulmonary disease

After transfer to our facility, the patient continued to have fevers and worsening shortness of breath. CXR again showed bilateral heterogeneous opacities (Fig. 1). Initial lab studies demonstrated a mild acidosis, elevated liver transaminases, hypoalbuminemia, increased international normalized ratio, and elevated inflammatory markers (Table 1). His respiratory viral panel, erythrocyte sedimentation rate (ESR), urine analysis, blood culture, and complete blood count were unremarkable. Pediatric infectious disease specialist was consulted, and a thorough infectious disease workup was performed (Table 1). Within 2 days, the patient developed acute respiratory decompensation prompting transfer to the pediatric intensive care unit and escalation of respiratory support to bilevel positive airway pressure (BiPAP).

**Table 1:** Initial lab studies obtained upon arrival to our institution

Lab test	Value	Reference range
White blood cell count	7.2	3.8–9.8 k/µL
Red blood cell count (RBC)	4.75	4.03–5.29 m/µL
Hemoglobin	12.3	11.0–14.5 g/dL
Hematocrit	38.0	33.9–43.5%
Mean corpuscular volume	80.0	77.0–89.0 fL
Mean corpuscular hemoglobin	25.9	25.2–30.2 pg
Mean corpuscular hemoglobin concentration	32.4	31.8–34.8 g/dL
Red cell distribution width	**15.1 (H)**	12.4–14.5%
Platelet count	240	175–332 k/µL
Neutrophils (%)	**83 (H)**	33–75%
International normalized ratio	**1.39 (H)**	0.9–1.1
Sodium	137	136–145 mmol/L
Potassium	3.8	3.5–5.1 mmol/L
Chloride	**109 (H)**	98–107 mmol/L
Total CO_2_	**20 (L)**	21–32 mmol/L
Anion gap	8	5–14 mmol/L
Blood urea nitrogen	5	5–18 mg/dL
Creatinine	**0.46 (L)**	0.70–1.30 mg/dL
Glucose	**106 (H)**	65–99 mg/dL
Serum osmolality	282	268–292 mOsm/kg
Calcium	**7.8 (L)**	8.5–10.1 mg/dL
Total protein	6.1	6.0–8.0 g/dL
Albumin	**2.9 (L)**	3.0–5.4 g/dL
Globulin	3.2	2.4–4.4 g/dL
Albumin/Globulin ratio	0.9	0.7–2.5 ratio
Prealbumin	**9 (L)**	20–40 mg/dL
Bilirubin, total	0.4	0.2–1.0 mg/dL
Alanine aminotransferase	43	16–61 U/L
Aspartate aminotransferase	**84 (H)**	15–37 U/L
Alkaline phosphatase	149	0–389 U/L
Gamma-glutamyl transferase	**137 (H)**	0–44 U/L
C-reactive protein	**3.0 (H)**	0.0–0.9 mg/dL
Erythrocyte sedimentation rate Westergren	10	3–13 mm/hour
Ferritin	**483 (H)**	26–388 ng/mL

Abnormal values noted in bold. Additional infectious disease testing that resulted negative or normal were as follows: *Mycobacterium tuberculosis* polymerase chain reaction (PCR) × 2, acid fast bacteria sputum × 3, purified protein derivative (PPD), stool PCR panel, stool ova and parasite, hepatitis panel, human immunodeficiency virus, respiratory sputum culture (normal flora only), *legionella* antibody, *bartonella* antibody, *blastomycose* antibody, *mycoplasma* antibody, *coccidioides* antibody, *brucella* antibody, cytomegalovirus antibody, *leptospirosis* antibody, *rickettsia typhi* antibody, nares swab for Methicillin-resistant *Staphylococcus aureus*, and urine streptococcal antigen. Interferon-γ release assay (QuantiFERON-TB Gold) returned indeterminate (due to high Nil > 8)

**Fig. 2:**
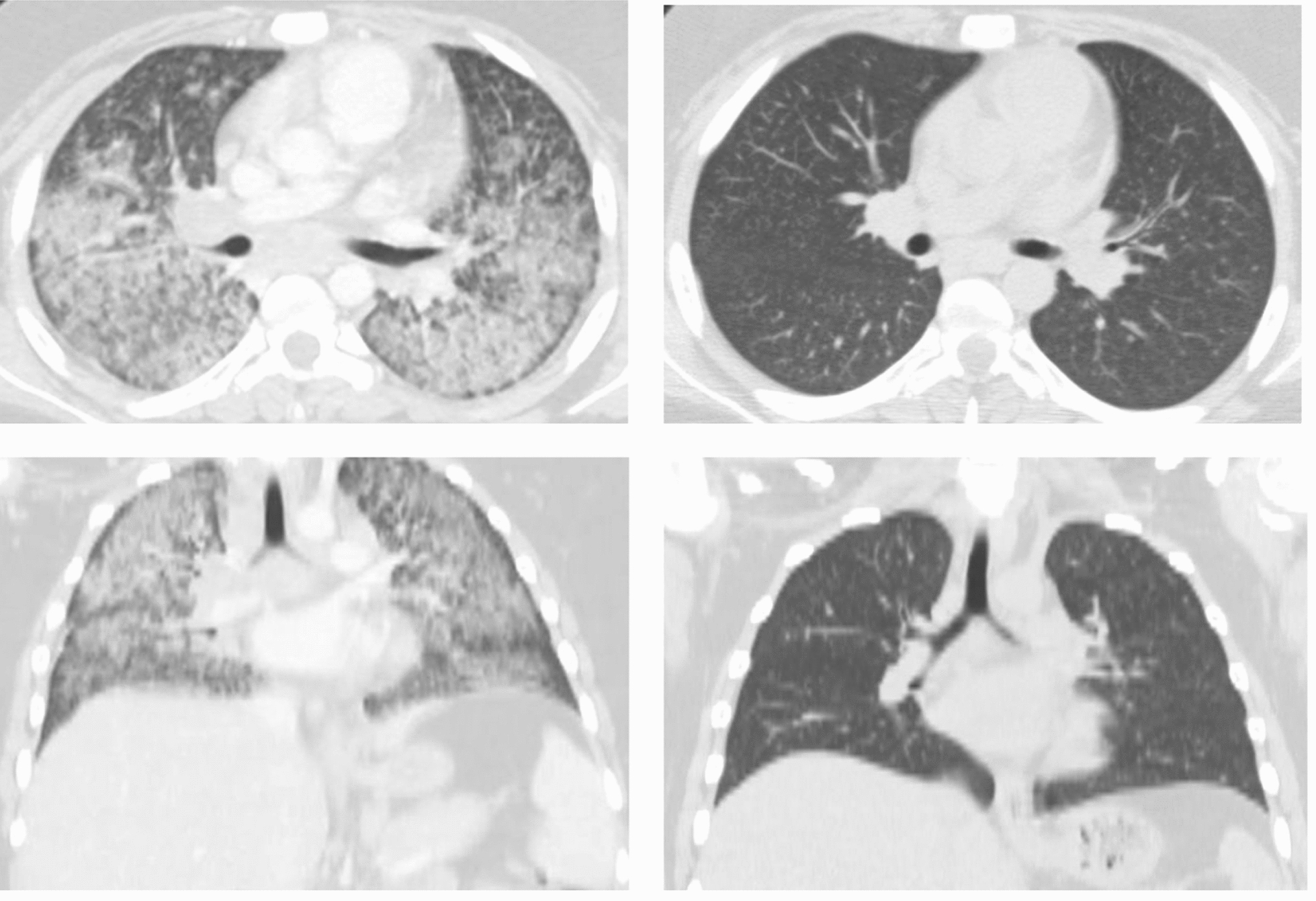
Computed tomography (CT) of the chest in axial (top) and coronal (bottom) planes. On admission, initial CT (left) showing extensive pulmonary disease. Repeat CT (right), obtained after inpatient liposomal amphotericin B and outpatient posaconazole therapy were completed, showing resolution of the infiltrative pulmonary disease

Computed tomography (CT) of the chest showed diffuse pulmonary infiltrates with slight nodular appearance and mild mediastinal and hilar adenopathy (Fig. 2, Supplementary Figs. 1 and 2). Abdominal ultrasound demonstrated a mildly echogenic and enlarged liver with mild distention of the common bile duct without gallstones. The patient continued to require significant oxygen support, so rifampin/isoniazid/pyrazinamide/ethambutol, azithromycin, and fluconazole were started empirically for tuberculosis (TB), atypical pneumonia, and fungal coverage, respectively. Given worsening inflammatory markers, respiratory status, and pulmonary infiltrates on CXR, there was concern for acute respiratory distress syndrome. Antimicrobial coverage was expanded to cefepime, vancomycin, and liposomal amphotericin B, and pulse dose steroids were initiated with IV methylprednisolone.

*Histoplasma* urine antigen eventually returned positive (>24.0 ng/mL) and given negative preliminary TB studies (Table 1), all antibacterial therapy was discontinued. A commercially available test for cell-free pathogen DNA using next generation sequencing (Karius test®, Karius Laboratory, Redwood City, CA), returned positive for *Histoplasma capsulatum* (5954 DNA molecules per uL), confirming the diagnosis of histoplasmosis. The patient’s GI symptoms resolved over the next few days of therapy, and he no longer required oxygen by hospital day 11. He completed a 2-week course of inpatient amphotericin B and was transitioned to oral posaconazole (300 mg daily) prior to discharge to complete a total 12-week course of antifungal therapy. After completion of his antifungal course, he demonstrated complete clinical and radiographic (Fig. 2) resolution of his histoplasmosis, as well as negative follow-up *Histoplasma *urine antigen testing. At the time of submission of this manusript, he had remained clinically and radiographically free of recurrence for several months.

## Discussion

### A note about histoplasmosis treatment

*Histoplasma* exposure rarely leads to symptomatic illness in healthy persons, and approximately 95% of individuals are asymptomatic after exposure [16]. The development of histoplasmosis and degree of severity are dependent on the extent of inoculum exposure in addition to other factors such as immune status and genetic susceptibility [16, 18, 19]. Treatment also varies greatly depending on disease severity and immune status of the patient. For immunocompetent patients, mild-to-moderate disease of less than 4 weeks can be managed symptomatically without the need for antifungal therapy [3, 18]. In those who have persistent symptoms after 4 weeks, a loading dose followed by a 6–12-week course of itraconazole (ITZ) is recommended [3, 18, 19]. For acute diffusesevere pulmonary disease, a 2-week course of amphotericin B, preferably the liposomal formulation which has reduced nephrotoxicity, should be implemented followed by the above ITZ regimen [3, 19]. For patients that are immunocompromised, ITZ may be implemented for exposures given the increased likelihood of developing severe disease [3, 18]. Due to availability or patient tolerance, posaconazole, voriconazole, or isavuconazole may be used as alternatives to ITZ [3, 18]. Of note, this class of triazoles can inhibit 11β-HSD2 and lead to apparent mineral corticoid excess manifesting as hypertension and hypokalemia, which were observed in our patient [20–22].

### Redefining geographic risk for histoplasma

**Fig. 3:**
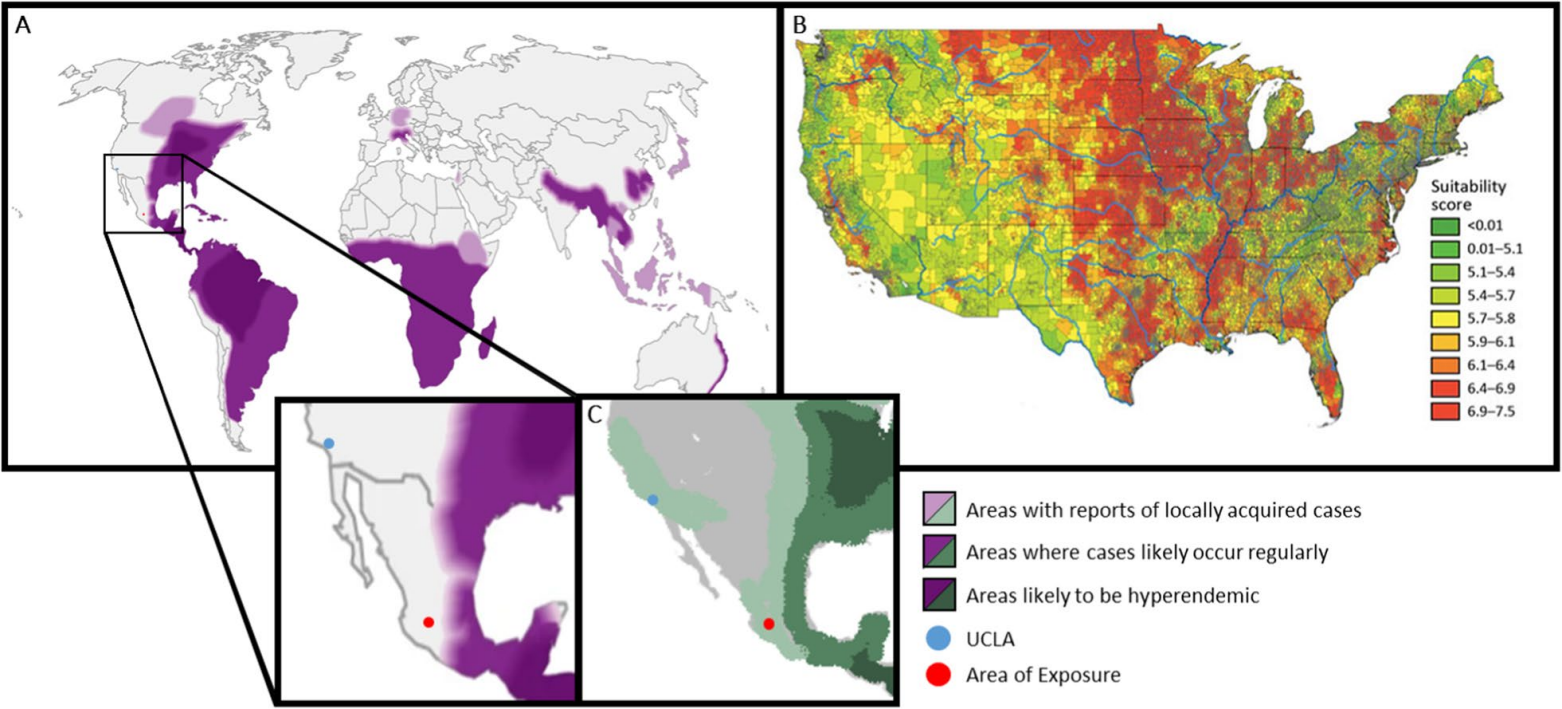
**A** Current worldwide *Histoplasma* endemicity estimates endorsed by the Centers for Disease Control and Prevention [24]. **B** Statistical modeling estimates of fungal prevalence based on environmental conditions [25]. **C** Newer estimates proposed by Barros *et al*. [18]. *Reused and edited under Creative Commons Attribution 4.0 International License with additions of sites for our institution (blue) and the location of exposure (red)

Histoplasmosis is often linked to excrement exposure from birds, chickens, or bats and is classically described after spelunking activities [23]. Epidemiologic studies have expanded our understanding of inoculum exposure to include fertilizer applications (particularly those made with bat guano), poultry farming, areas near heavy bird excrement, and construction zones [4, 5]. Geographically, *Histoplasma* has traditionally been considered a global fungus with well-defined regional hot spots (Fig. 3A), but recent reports suggest additional areas should be considered high risk.

Currently, the central and eastern parts of the USA are designated as endemic, traditionally described as the Ohio and Mississippi River valleys [26]. Estimation of the incidence of histoplasmosis in the USA is difficult as only ten states submit incidence data to the National Notifiable Diseases Surveillance System (Rhode Island, Pennsylvania, Delaware, Michigan, Wisconsin, Minnesota, Kentucky, Illinois, and Louisiana), while only four additional states mandate cases be reported at the local/state level (Arkansas, Indiana, Kansas, and Nebraska) [27]. Case reports and epidemiologic data from the past two decades have revealed western and northern expansions of *Histoplasma*, particularly along the Missouri River Valley [28–31]. This has led to a newer, CDC-endorsed model of at-risk regions along additional river valleys and smaller areas outside of traditional regions, notably Southern California (Fig. 3B) [25].

Understanding the incidence and prevalence patterns of histoplasmosis remains similarly challenging in Mexico, where it is not a reportable disease [32]. A report by the National Institute of Respiratory Diseases of Mexico identified cases in only 9 of the 32 Mexican states, [33] but cases of histoplasmosis are both increasing in low-endemic areas and expanding to new locations [33, 34]. Currently, the CDC only outlines areas of *Histoplasma* endemicity in the eastern half of Mexico, especially along the Gulf of Mexico, the southern region, and the Yucatan Peninsula (Fig. 3A) [24]. However, several case series and local epidemiologic data have surfaced over the last few decades outlining a growing concern for *Histoplasma* endemicity in central to western parts of Mexico and as far north as Sinaloa [15, 35–45].

One case report described a 58-year-old man who had visited Puerto Vallarta, Jalisco and developed symptoms one week after his return to the USA [38]. Another report described a 22-year-old Jalisco resident found to have epididymitis secondary to histoplasmosis [39]. A small case series described three temporary workers from Japan who developed pulmonary disease after visiting Manzanillo, Colima, an area southwest of Jalisco along the western coast [40]. Interestingly, these individuals spent most of their time at their hotel or workplace and had no typical exposure history such as spelunking. Review of other hotel guests failed to identify further cases of histoplasmosis, and thus, dusty factory conditions were presumed to be the source of the inoculum. Another case series described a large outbreak among US college students who had visited Acapulco in the state of Guerrero, further south of Jalisco and Colima [41]. These individuals had also not been involved with typical exposures such as spelunking, and their exposure likely occured in their hotel that was undergoing minor construction, specifically from a dirty and dusty stairwell. Ultimately, 147 individuals were identified as contracting histoplasmosis due to exposure within the hotel. There were other accounts of individuals in the area of the hotel who had contracted histoplasmosis. A subsequent outbreak later that year suggested fertilizer in the potting soil of the hotel’s plants could have also been responsible [42]. It should be noted that the city of Acapulco is significantly closer to the areas known to be endemic in the heat map (Fig. 3A) compared with the other reports described.

There are also reports in the human immunodeficiency virus (HIV) literature describing histoplasmosis in locations outside of the CDC-identified zones [43–46]. For example, a small case series of persons with HIV from Guadalajara showed successful treatment with ITZ for their histoplasmosis infection [47]. This population may be a better surrogate of *Histoplasma* endemicity given that the development of symptomatic disease occurs more readily and at a lower inoculum. Medical centers in Jalisco treat dozens of immunocompromised patients for disseminated histoplasmosis each year, and histoplasmosis remains one of the most common opportunistic infections and leading cause of hospitalization and death among patients with acquired immunodeficiency syndrome (AIDS) (per private communications with local infectious disease experts). Inoculation is thought to result from bird and bat guano exposure. Other local experts have also published histoplasmosis cases from outside the CDC-defined at-risk areas in Mexico [48].

Despite these findings and growing concerns from local experts, individual states (particularly in central and western Mexico) have not officially performed testing or updated regional endemicity of *Histoplasma* for several years [49]. A recent report by Barros *et al*. proposed a newer world map of *Histoplasma* incidence and incorporates more expansive areas at risk within Mexico and the USA [18]. Given the findings of our literature search and expert opinions from local infectious disease physicians, we believe the Barros *et al*. map more accurately reflects the true endemicity of *Histoplasma* in Western Mexico. We are advocating for a revision to the current CDC-endorsed map to not only include these changes but also to consider the growing literature describing cases from other areas around the world, such as North America, India, and Southeast Asia [18, 50, 51].

## Conclusion

Histoplasmosis remains a global problem, and areas of potential inoculum exposure appear to be expanding [25, 52, 53]. Advancing population growth, environmental disruption, farming, climate change, travel, commercialization, growing prevalence of immunocompromised populations, and enhanced testing may all be influencing increased case detection and reporting [18, 23]. Local experts are aware of these changing incidence patterns. However, histoplasmosis does not always fall under mandatory reporting guidelines, making readily available data from many areas scarce [54, 55] and resulting in delays in updating global infectious disease recommendations. Accurate estimation of high-risk geographies both within the USA and abroad are important for local and international practicioners to make recommendations about prevention strategies, especially for immunocompromised patients in whom such an infection could prove fatal [56, 57]. In the pursuit of understanding this patient’s inoculum exposure, a review of the literature revealed an apparent lack of global recognition for central and western Mexico’s histoplasmosis incidence rates, despite known cases being reported and soil assessment confirming the presence of *Histoplasma* since the 1980s [58, 59]. Despite increasing cases and growing concerns from local experts, reevaluations of individual Mexican states’ *Histoplasma* status, particularly in the central and western regions, have not been performed for several years [49]. A recent report by Mexico’s National Institute of Respiratory Diseases acknowledges these growing concerns, and the prevalence of *Histoplasma* may now be extending to areas previously thought to be of minimal risk for this infectious agent [33]. Given these data, we advocate for a revision to the current CDC-endorsed maps and expansion to additional worldwide regions in conjunction with the new report by Barros *et al.* [18].

## Supplementary Information

Supplementary Material 1

## Data Availability

Supplementary Fig. 1: Axial chest CT displaying pulmonary disease; Supplementary Fig. 2: Coronal chest CT displaying pulmonary disease.
