# Supplementary material for: Disseminated histoplasmosis from western Mexico—rethinking our geographic distribution of endemic fungal species: a case report and review of literature
Source: J Med Case Rep. 2024 Nov 8;18:540. doi: 10.1186/s13256-024-04856-x (PMC11545083; doi:10.1186/s13256-024-04856-x)
Supplement: Supplementary file 1 — Supplementary Material 1 [file 13256_2024_4856_MOESM1_ESM.pptx]

## Slide 1
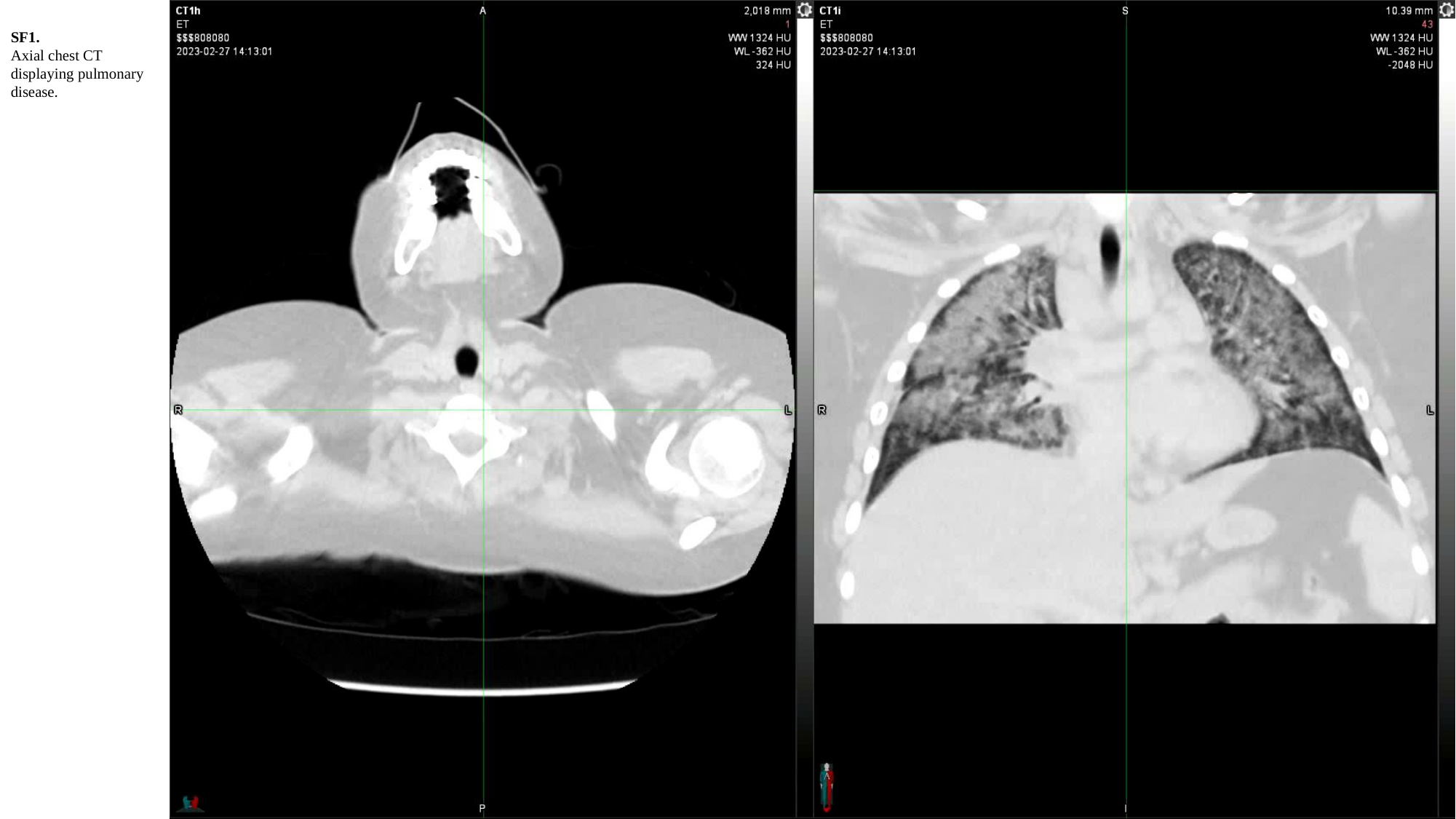

SF1.
Axial chest CT displaying pulmonary disease.

## Slide 2
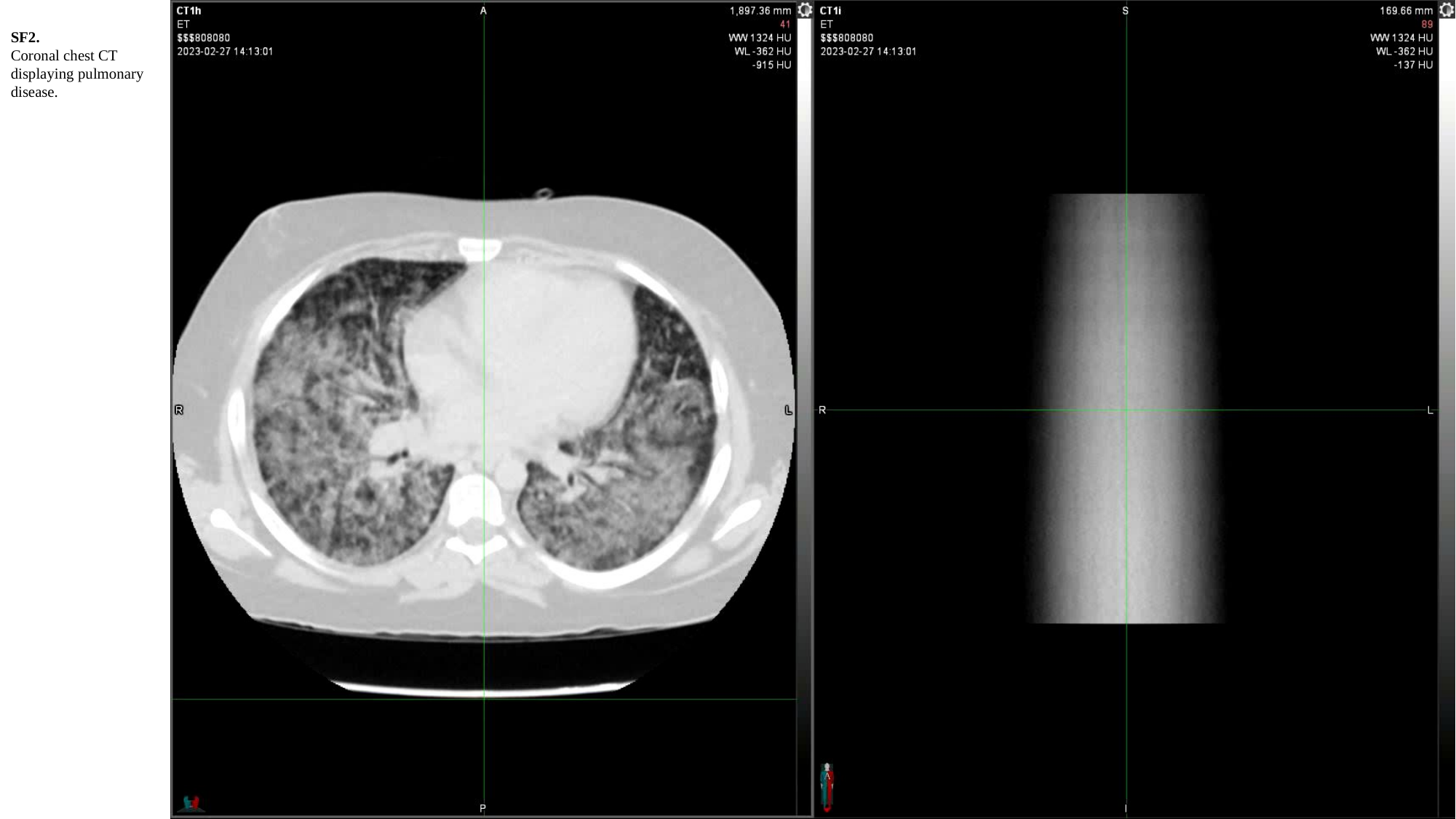

SF2.
Coronal chest CT displaying pulmonary disease.
